# Adherence to dietary guidelines and risk of dementia: a prospective cohort study of 94 184 individuals

**DOI:** 10.1017/S2045796022000567

**Published:** 2022-10-10

**Authors:** E. W. Kjeldsen, J. Q. Thomassen, K. L. Rasmussen, B. G. Nordestgaard, A. Tybjærg-Hansen, R. Frikke-Schmidt

**Affiliations:** 1 Department of Clinical Biochemistry, Copenhagen University Hospital – Rigshospitalet, Blegdamsvej 9, 2100 Copenhagen, Denmark; 2 Department of Clinical Medicine, Faculty of Health and Medical Sciences, University of Copenhagen, Blegdamsvej 3, 2200 Copenhagen, Denmark; 3 The Copenhagen General Population Study, Copenhagen University Hospital – Herlev and Gentofte, Borgmester Ib Juuls Vej 1, 2730 Herlev, Denmark; 4 Department of Clinical Biochemistry, Copenhagen University Hospital – Herlev and Gentofte, Borgmester Ib Juuls Vej 1, 2730 Herlev, Denmark

**Keywords:** Ageing, diet, lipids, non-Alzheimer's dementia

## Abstract

**Aims:**

Recent estimates suggest that 40% of dementia cases could be avoided by treating recognised cardiovascular risk factors such as hypertension, diabetes, smoking and physical inactivity. Whether diet is associated with dementia remains largely unknown. We tested if low adherence to established dietary guidelines is associated with elevated lipids and lipoproteins and with increased risk of Alzheimer's disease and non-Alzheimer's dementia – a dementia subtype with a high frequency of cardiovascular risk factors.

**Methods:**

We used the prospective Copenhagen General Population Study including 94 184 individuals with dietary information and free of dementia at baseline. Mean age at study entry was 58 years, and 55% (*N* = 51 720) were women and 45% (*N* = 42 464) were men. Adherence to dietary guidelines was grouped into low, intermediate and high adherence based on food frequency questionnaires. Main outcomes were non-Alzheimer's dementia and Alzheimer's disease.

**Results:**

Low-density lipoprotein cholesterol, non-high-density lipoprotein cholesterol and plasma triglyceride levels were higher in individuals with intermediate and low adherence to dietary guidelines compared with individuals with high adherence (all *p* for trends <0.001). Age and sex-adjusted hazard ratios (HRs) for non-Alzheimer's dementia *v.* individuals with high adherence were 1.19 (95% confidence interval 0.97–1.46) for intermediate adherence, and 1.54 (1.18–2.00) for low adherence. Corresponding HRs in multivariable-adjusted models including *APOE* genotype were 1.14 (0.92–1.40) and 1.35 (1.03–1.79). These relationships were not observed in individuals on lipid-lowering therapy.

**Conclusions:**

Low adherence to national dietary guidelines is associated with an atherogenic lipid profile and with increased risk of non-Alzheimer's dementia – the subtype of dementia with a high frequency of vascular risk factors. This study suggests that implementation of dietary guidelines associated with an anti-atherogenic lipid profile could be important for prevention of non-Alzheimer's dementia.

## Graphical abstract

**Figure:**
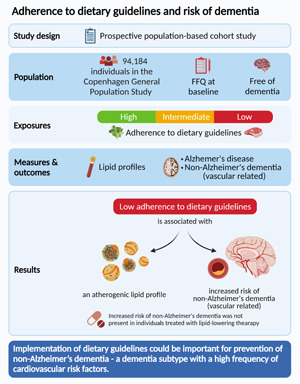

## Introduction

Dementia is a devastating disease currently affecting the activity of daily life in 50 million people (World Health Organization, 2017). Due to ageing of populations worldwide, the number is estimated to grow to 152 million by 2050 (World Health Organization, 2017). No curative treatment or medication to effectively halt dementia exist (World Health Organization, 2017). Therefore, it is of utmost importance to establish and act on modifiable risk factors to implement strategies to prevent or delay dementia onset. Approximately 40% of dementia cases are considered caused by modifiable vascular risk factors such as hypertension, diabetes, smoking and physical inactivity. This leaves a substantial potential for prevention (Livingston *et al*., 2020), where diet could play a central role in reducing occurrence of vascular risk factors. Although the contribution of diet to death is recognised to be substantial globally (Abbafati *et al*., 2020), the association with dementia remains largely unknown (Livingston *et al*., 2020).

Recently, the Global Burden of Disease report showed that dietary risk was a major contributor to attributable deaths (Abbafati *et al*., 2020). Additionally diet quality was the fifth leading risk factor for disability due to cardiovascular disease, diabetes, kidney diseases and neoplasms (Abbafati *et al*., 2020). Furthermore, current diets in most countries worldwide are too unhealthy and dietary changes as recommended by national guidelines could lower rates of premature mortality (Springmann *et al*., 2020). Adherence to a healthy diet could therefore have a promising potential for improving public health but also for preventing dementia. A systematic review concluded that a Mediterranean diet may contribute to better cognitive function, but that there is a need for larger epidemiological studies taking more factors such as the presence of comorbidities into account (Petersson and Philippou, 2016). Furthermore, adherence to a healthy diet by mainly using unsaturated fat, eating fruit and plenty of vegetables, eating more fish, choosing whole grain foods and consuming only moderate amounts of sugar and foods high in salt content, is associated with lower risk of the dementia subtype Alzheimer's disease (Féart *et al*., 2009; Morris *et al*., 2015; Haring *et al*., 2016; Van Den Brink *et al*., 2019). Individuals with vascular and unspecified dementia have a high frequency of cardiovascular risk factors. These diagnoses are often grouped into a combined dementia subtype, called non-Alzheimer's dementia. It is, however, unknown whether diet is associated with this type of dementia (Perez *et al*., 2012; Wu and Sun, 2017; Akbaraly *et al*., 2019; Barbaresko *et al*., 2020; McKenzie *et al*., 2021). Consequently, it is important to explore the gaps in knowledge and examine the association of adherence to established dietary guidelines with risk of non-Alzheimer's dementia and Alzheimer's disease in a large population-based study with long follow-up.

The aim of this study was to investigate the relationship between adherence to established dietary guidelines and lipids and lipoproteins and risk of Alzheimer's disease and non-Alzheimer's dementia. Adherence to established dietary guidelines was assessed by a simple food frequency questionnaire in a large prospective cohort, The Copenhagen General Population Study (CGPS), including 94 184 individuals aged 20–100 years at baseline.

## Materials and methods

The study was approved by institutional review boards and a Danish Ethical Committee (H-KF-01-144/01) and was conducted according to the Declaration of Helsinki (revision 2000). All individuals were white and of Danish descent. Written informed consent was obtained from all individuals.

### Participants

The CGPS was initiated in 2003–2015 (online Supplementary Figs S1 and S2) with follow-up examinations starting in 2015 (CGPS2) (Jørgensen *et al*., 2014; Rasmussen *et al*., 2019; Juul Rasmussen *et al*., 2020). To reflect the adult Danish population aged 20–100 years, individuals were selected randomly based on the Danish Civil Registration System. Data were obtained at baseline (study inclusion) from a self-administered questionnaire reviewed together with an investigator on the day of attendance, a physical examination and from blood samples including DNA extraction. Information on diet, vital status and disease status was available for 94 184 individuals who were followed for a median of 9 years. A table of baseline characteristics for individuals included and excluded from this study is provided in online Supplementary Table S1. Individuals participating in the study were by linkage to the Danish registries followed with information on diagnosis codes, emigration status and information from the causes of death registry. We excluded 137 individuals with a dementia diagnosis at baseline.

### Dietary assessment and dietary categories

Dietary habits were assessed from a short food frequency questionnaire (FFQ) which was a part of the extensive overall questionnaire used in CGPS (online Supplementary Table S2). The short FFQ was developed in collaboration with nutrition specialists and included important items based on the Danish dietary guidelines to assess overall dietary habits (Ewers *et al*., 2021). This kind of categorisation showed strong associations with cardiovascular disease and mortality (Ewers *et al*., 2021). The FFQ focused on selected key items of the Danish food-based dietary guidelines, i.e. the recommendations to: (1) eat less saturated fat, (2) eat fruit and plenty of vegetables, (3) eat more fish, (4) eat less sugar and (5) eat foods with less salt (The Official Dietary Guidelines – Good for Health and Climate, 2020). The FFQ specifically explored: (1) dietary fat quality in cold and warm meals (saturated fats: butter, butter-based blends and hard margarines; unsaturated fats: soft margarines and vegetable oils); (2) usual intake frequencies (from almost never to several daily servings) of fruit, vegetables, fish, sucrose-sweetened beverages, cold meat cuts like sausages and pâtés for open sandwiches and fast food (Ewers *et al*., 2021). The FFQ did not cover intake of foods directly related to the remaining recommendations of the Danish food-based dietary guidelines advocating to choose whole grain foods, to choose low-fat dairy products, to eat low-fat meat and meat products or to drink water. Nor did the FFQ include assessment of portion sizes.

For consistency between studies, we used an identical set-up as in a previous study that used the same short FFQ; we classified our FFQ questions into three levels of importance (from A to C) for an overall healthy dietary pattern (Ewers *et al*., 2021). Class A questions focused on major contributors to the dietary macronutrient composition, specifically dietary fat quality (fats in cold and warm meals: unsaturated *v.* saturated fat) and dietary fibre content (fruit: ⩾3 *v*. <3 weekly servings and vegetables: ⩾3 *v*. <3 weekly servings). Class B questions elucidated intake of specific foods considered healthy (fish: ⩾3 *v*. <1 weekly servings), or unhealthy (sugar sweetened beverages: <0.5 *v*. ⩾1 l/week). Class C questions focused on foods rich in salt (cold meat cuts like sausages and pâtés for open sandwiches: <5 *v*. ⩾7 weekly servings and fast foods: <1 *v*. ⩾1 weekly servings) (Ewers *et al*., 2021).

**Fig. 1.**
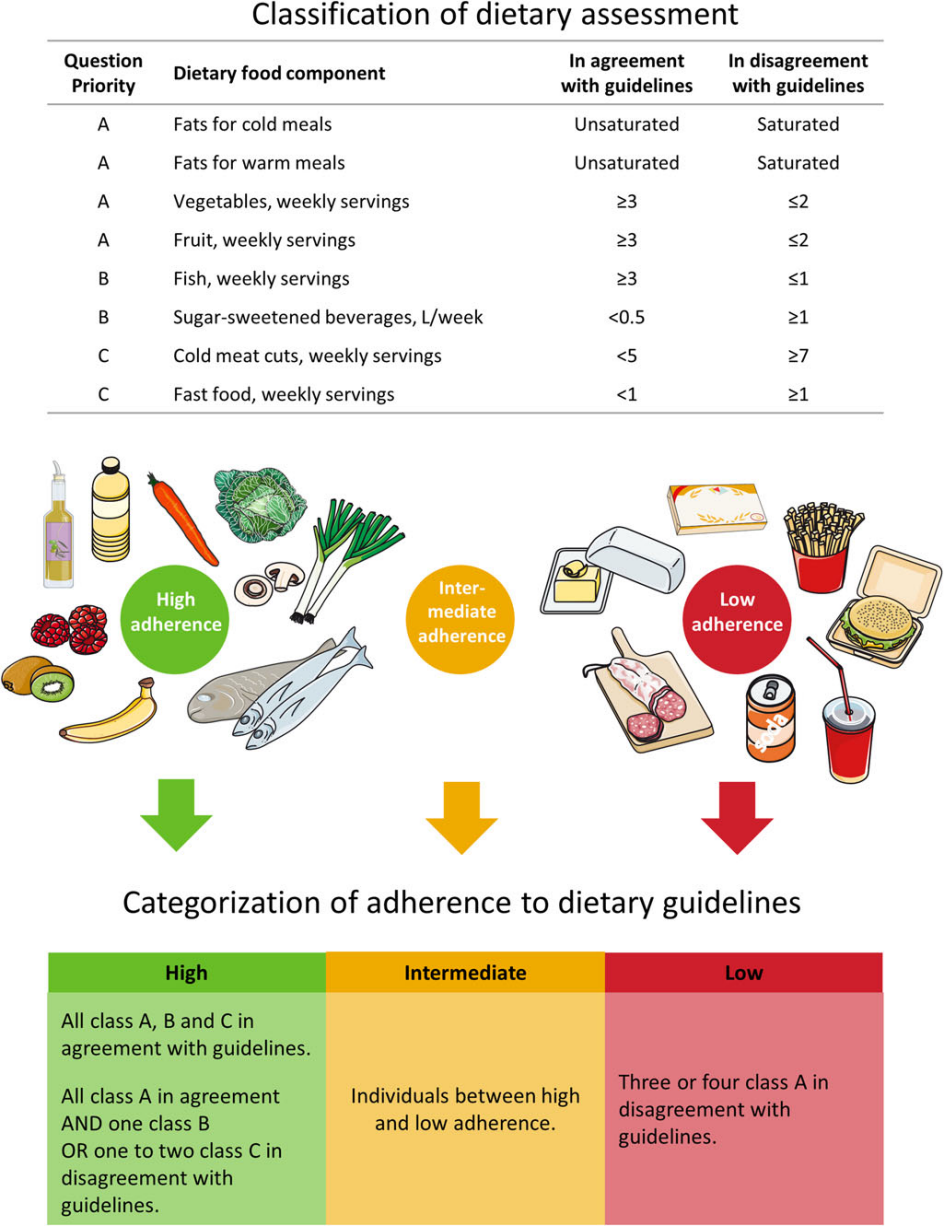
Dietary assessment in 94 184 individuals from the CGPS. FFQ questions were classified into three levels of importance (from A to C) according to an overall healthy dietary pattern. Based on the FFQ, individuals were divided into three predefined categories ranging from high to low adherence to current dietary guidelines. See ‘Methods’ for further details. FFQ, Food Frequency Questionnaire.

Based on the FFQ, individuals were divided into three predefined categories ranging from high to low adherence to current dietary guidelines. High adherence: all class A, B and C answers in agreement with current guidelines; or all A, B and C answers in agreement with current guidelines except for either one class B answer in disagreement with guidelines or one or two class C answers in disagreement with guidelines. Intermediate adherence: individuals between high and low adherence categories. Low adherence: three or four class A answers in disagreement with current guidelines (Fig. 1 and online Supplementary Table S3).

We included individuals participating in both CGPS and CGPS2 (*n* = 17 506) in an analysis to estimate changes in dietary patterns from baseline to second examination follow-up. A total of 12 800 individuals filled out FFQs at both CGPS and CGPS2. The FFQ included the same questions at both visits. The median time interval between first examination and follow-up was 10 years.

Description of clinical endpoints, laboratory analyses, genotyping, covariates and statistical analyses is provided in the online Supplementary materials, Methods.

## Results

Baseline characteristics of 94 184 individuals stratified by three groups of adherence to dietary guidelines are shown in Table 1. The mean age of participants at study inclusion was 58. Fifty-five per cent (51 720/94 184) were women and 45% (42 464/94 184) were men. Analyses were conducted in men and women combined as no interaction between sex and exposure in the association with dementia was observed (*p*-value = 0.12). Twenty-one per cent of individuals had high adherence to dietary guidelines, 70% had intermediate adherence and 9% had low adherence. Individuals in the group with low adherence to dietary guidelines were more frequently men, had shorter education, lower household income, higher body mass index, were less physically active, had a higher tobacco and alcohol consumption, had more frequently hypertension, and treatment with lipid-lowering therapy was less frequent.

**Table 1.** Baseline characteristics of 94 184 individuals grouped according to degree of adherence to Danish dietary guidelines

	Adherence to dietary guidelines		
Baseline characteristics (*N* = 94 184)	High	Intermediate	Low
Number, no. (%)	19 679 (20.9)	65 903 (70.0)	8602 (9.0)
Age, years (median, IQR)	59.6 (50.8–67.4)	57.3 (47.4–67.0)	60.1 (49.0–70.2)
Men, no. (%)	6592 (33.5)	30 222 (45.9)	5650 (65.7)
Low household income, no. (%)	6177 (31.4)	22 706 (34.5)	4483 (52.1)
Education <8 years, no. (%)	1431 (7.3)	5839 (8.9)	1506 (17.5)
Body mass index, kg/m^2^ (median, IQR)	25.4 (23.1–28.1)	25.5 (23.2–28.4)	(23.5–29.1)
Physical inactivity in leisure time, no. (%)	7925 (40.3)	31 732 (48.2)	5542 (64.4)
Cumulative tobacco consumption, pack-years (median, IQR)	12 (5–24)	15 (6–30)	29 (15–44)
High alcohol consumption, no. (%)	3215 (16.3)	11 055 (16.8)	1945 (22.6)
Hypertension, no. (%)	11 917 (60.6)	39 161 (59.4)	5669 (65.9)
Diabetes mellitus, no. (%)	935 (4.8)	2592 (3.9)	409 (4.8)
Lipid-lowering therapy, no. (%)	2999 (15.2)	7477 (11.4)	1009 (11.7)

IQR, interquartile range.

Low education corresponds to completion of primary school or less. See Methods (covariates) in the online Supplementary materials for details.

### Changes in diet over time

The frequency of individuals who did not change their diet was 8382 individuals out of 12 800 (65%). A total of 4336 (34%) individuals changed their diet to either one group higher or lower in adherence to dietary guidelines, whereas only 72 individuals (0.6%) changed two groups: from high to low or from low to high adherence to dietary guidelines. The specific number of individuals for each diet group either increasing or decreasing their adherence to dietary guidelines is detailed in online Supplementary Table S5. The distribution of individuals in the three dietary categories was largely similar at baseline and at follow-up indicating no major changes in overall diets.

### Lipids and lipoproteins

**Fig. 2.**
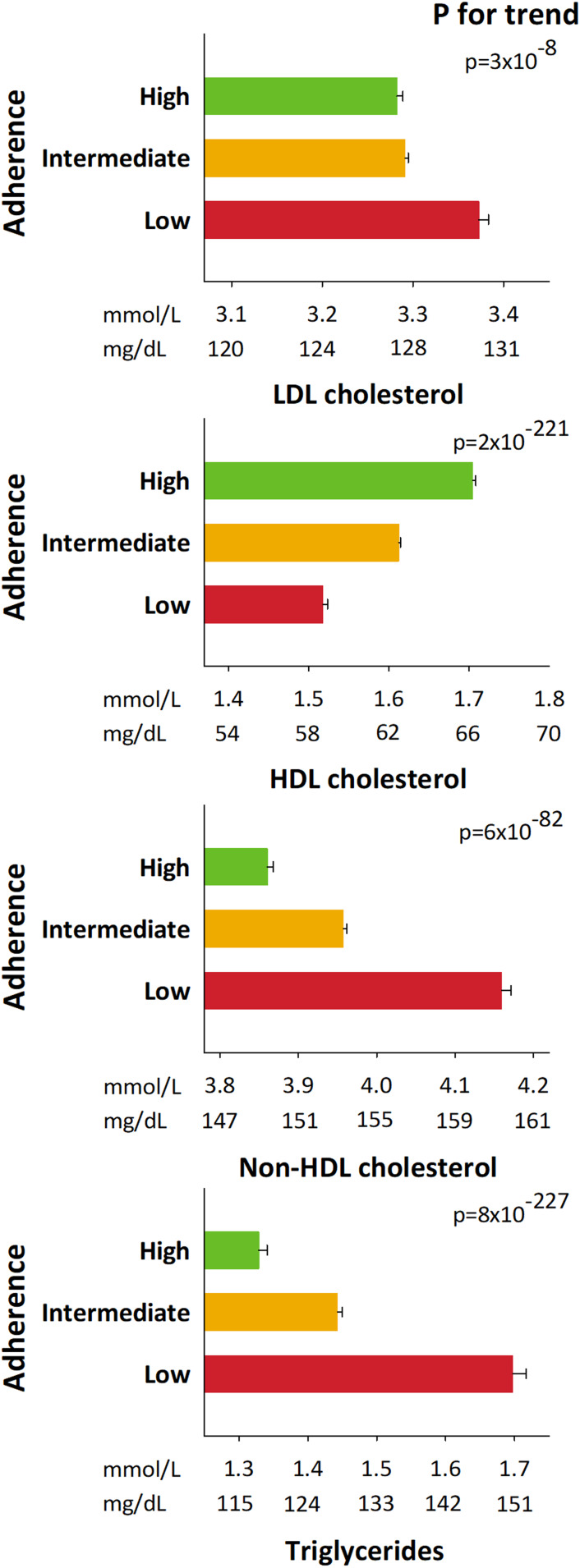
Plasma levels of lipids and lipoproteins as a function of adherence to dietary guidelines in 94 184 individuals from the CGPS. Geometric mean ± standard errors of the mean are given for triglycerides; arithmetic mean ± standard errors of the mean are given for LDL cholesterol, HDL cholesterol and non-HDL cholesterol. LDL cholesterol, low-density lipoprotein cholesterol; HDL cholesterol, high-density lipoprotein cholesterol.

Levels of low-density lipoprotein (LDL) cholesterol, non-high-density lipoprotein (HDL) cholesterol and plasma triglycerides increased stepwise for individuals from high over intermediate to low adherence to dietary guidelines, while HDL cholesterol levels decreased (*p*-values ranging from 8 × 10^−227^ to 3 × 10^−8^) (Fig. 2).

### Risk of dementia

**Fig. 3.**
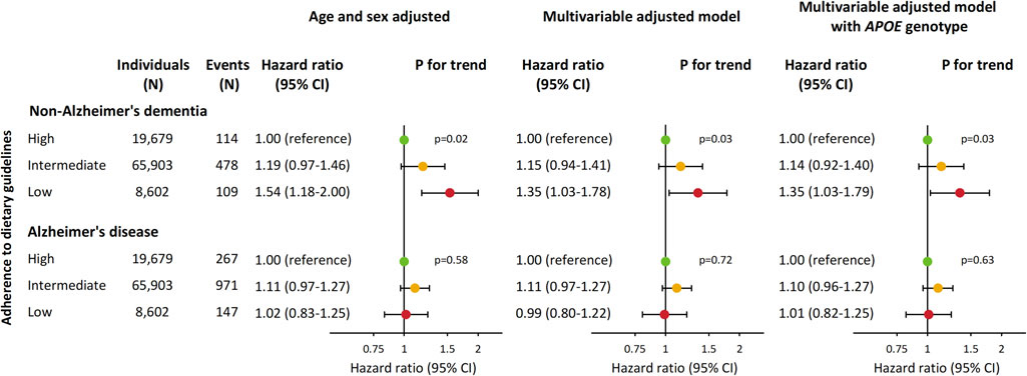
Risk of non-Alzheimer's dementia and Alzheimer's disease according to dietary groups in 94 184 individuals from the CGPS. Multivariable adjustment was for age, sex, household income, education, pack-years, alcohol consumption, physical activity, body mass index, diabetes, hypertension, lipid-lowering therapy, LDL cholesterol, HDL cholesterol and triglycerides. *APOE* genotype, apolipoprotein E *ɛ*2/*ɛ*3/*ɛ*4 genotype; CI, confidence interval; HDL cholesterol, high-density lipoprotein cholesterol; LDL cholesterol, low-density lipoprotein cholesterol.

Age and sex-adjusted hazard ratios (HRs) for non-Alzheimer's dementia increased stepwise with lower adherence to dietary guidelines (Fig. 3). Compared with individuals with high adherence, age and sex-adjusted HRs were 1.19 (95% confidence interval 0.97–1.46) for individuals with intermediate adherence and 1.54 (1.18–2.00) for individuals with low adherence. Corresponding multivariable-adjusted HRs were 1.15 (0.94–1.41) and 1.35 (1.03–1.78) while corresponding HRs when further adjusting for *APOE* genotype were 1.14 (0.92–1.40) and 1.35 (1.03–1.79). In contrast, adherence to dietary guidelines was not associated with risk of Alzheimer's disease. Results were similar for both non-Alzheimer's dementia and Alzheimer's disease in a sensitivity analysis where individuals with less than 2 years of follow-up were excluded (online Supplementary Fig. S3). Furthermore, when additionally adjusting for ischaemic heart disease and ischaemic cerebrovascular disease, or when using imputed variables based on all covariates and endpoints, results were similar (online Supplementary Figs S4 and S5).

### Cardiovascular and all-cause mortality as positive controls

Age and sex-adjusted HRs for cardiovascular mortality *v*. individuals with high adherence to dietary guidelines were 1.21 (1.07–1.38) for intermediate and 1.95 (1.67–2.28) for low adherence (online Supplementary Fig. S6). Corresponding HRs for all-cause mortality were 1.26 (1.19–1.33) and 1.97 (1.83–2.12), respectively. Results were similar after multivariable adjustment.

### Interaction with lipid-lowering therapy

**Fig. 4.**
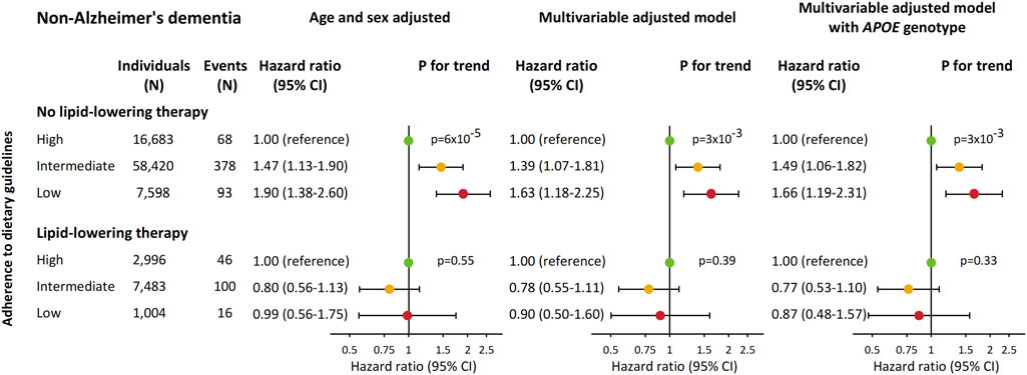
Risk of non-Alzheimer's dementia according to dietary groups stratified by lipid-lowering therapy in 94 184 individuals from the CGPS. Multivariable adjustment was for age, sex, household income, education, pack-years, alcohol consumption, physical activity, body mass index, diabetes, hypertension, LDL cholesterol, HDL cholesterol and triglycerides. *APOE* genotype, apolipoprotein E *ɛ*2/*ɛ*3/*ɛ*4 genotype; CI, confidence interval; HDL cholesterol, high-density lipoprotein cholesterol; LDL cholesterol, low-density lipoprotein cholesterol.

The *p*-value for interaction between dietary groups and lipid-lowering therapy on risk of non-Alzheimer's dementia was 0.04. No other interactions were detected for the remaining covariates (all *p* > 0.05) (online Supplementary Fig. S7). Consequently, we performed an analysis stratified by lipid-lowering therapy (Fig. 4). For individuals who were not treated with lipid-lowering therapy multivariable-adjusted HRs for non-Alzheimer's dementia were 1.39 (1.07–1.81) in individuals with intermediate adherence and 1.63 (1.18–2.25) in individuals with low adherence to dietary guidelines. Corresponding HRs for individuals treated with lipid-lowering therapy were 0.78 (0.55–1.11) and 0.90 (0.50–1.60), respectively.

## Discussion

In this prospective cohort study of 94 184 individuals from the Danish general population, low adherence to national dietary guidelines was associated with an atherogenic plasma lipid profile and with increased risk of non-Alzheimer's dementia, an association that did not appear to be present in individuals treated with lipid-lowering therapy. These findings are novel and may be clinically important as they suggest that non-Alzheimer's dementia can potentially be prevented by well-established interventions such as adhering to national dietary guidelines and by lipid-lowering treatment.

To our knowledge this is the first study to show an association between low adherence to dietary guidelines and increased risk of non-Alzheimer's dementia in Denmark as well as globally. Previous cross-sectional or prospective studies investigating the association between diet and risk of dementia have primarily observed an association between a healthy diet and less cognitive decline or a lower risk of Alzheimer's disease (Féart *et al*., 2009; Morris *et al*., 2015; Haring *et al*., 2016; Van Den Brink *et al*., 2019). These studies often focused on single nutrients or foods, did not adjust for all known risk factors and did not take *APOE* genotype into account (Cao *et al*., 2016; Van Den Brink *et al*., 2019; Barbaresko *et al*., 2020; Limongi *et al*., 2020; Zhang *et al*., 2021). Evidence of an association with all-cause dementia has been inconsistent (Wu and Sun, 2017; Akbaraly *et al*., 2019; Van Den Brink *et al*., 2019; McKenzie *et al*., 2021), and findings on vascular dementia are sparse (Perez *et al*., 2012). Randomised controlled trials including dietary interventions show results in favour of a healthy diet with higher cognition, however, with no measures on hard endpoints such as Alzheimer's disease or vascular dementia (Martínez-Lapiscina *et al*., 2013; Valls-Pedret *et al*., 2015). Non-Alzheimer's dementia is a dementia subtype associated with cardiovascular risk factors; the present findings on low adherence to dietary guidelines and non-Alzheimer's dementia firmly support that diet may play an important role in vascular health including cerebrovascular health and dementia with vascular pathology. The World Health Organization states that a ‘healthy and balanced diet should be recommended to all adults’, as it has the potential to prevent cognitive decline both directly and through other potential risk factors or diseases (WHO Guidelines, 2019).

During the past few years dietary research has changed from focusing on the role of single nutrients or food items to the role of dietary patterns like the Mediterranean diet, the Dietary Approaches to Stop Hypertension (DASH) or the Mediterranean–DASH Intervention for Neurodegenerative Delay (MIND) diet (Pistollato *et al*., 2018). Previous studies have frequently investigated these specific diets and their associations with dementia, which are all characterised by a high intake of vegetables, fruits and unsaturated fats with modest amounts of alcohol and animal foods. In general, observational studies have found that these diets are associated with lower risk of Alzheimer's disease and less cognitive decline (Van Den Brink *et al*., 2019). The current study did not use one of these established methods to characterise dietary patterns, however, the dietary assessment focused on very similar aspects with only minor discrepancies.

Dietary risk is one of the leading causes of attributable deaths in the Global Burden of Disease 2019 report, and diet quality is the fifth leading risk factor for disability mainly due to cardiovascular disease, diabetes, kidney diseases and neoplasms (Abbafati *et al*., 2020). In addition, our study suggests an increased risk of non-Alzheimer's dementia by non-adherence to dietary guidelines. Interestingly, a study published in 2020 found that current diets in most countries worldwide are too unhealthy and that national dietary guidelines should be reformed and better implemented (Springmann *et al*., 2020). The study showed that dietary changes as those recommended by dietary guidelines could be associated with substantially reduced premature mortality (Springmann *et al*., 2020). This would be by following the Mediterranean diet, the DASH, the MIND diet or more specifically by increasing consumption of healthy foods such as fruits, vegetables and legumes; reducing consumption of unhealthy foods, such as sugar, unrefined grains, salt, highly processed foods and red meat and choosing unsaturated fats rather than saturated fats (Willett *et al*., 2019). There are numerous ways of implementing food-based dietary guidelines e.g. by updating policies to incentivise better adherence to dietary guidelines for instance by investment in health promotion campaigns and programmes, regulation of agricultural strategies, changes in consumer behaviour, educating families on healthy diets and taxation of unhealthy food items (Sassi *et al*., 2018; Willett *et al*., 2019; Springmann *et al*., 2020). Furthermore, as this study shows a clear clustering of unfavourable cardiovascular risk factors in individuals with low adherence to dietary guidelines the potential to target health strategies towards specific subpopulations is evident. Moreover, updating national food-based dietary guidelines could be beneficial for meeting global sustainability goals (Springmann *et al*., 2020) in line with the EAT Lancet Commission and the United Nations Sustainable Development Goals 2030 Agenda (United Nations; Willett *et al*., 2019). Adopting a sustainable diet rich in plant-based foods, low in animal source foods and highly processed foods would not only benefit human health but also the environment (Willett *et al*., 2019). A huge potential for prevention exists, unfortunately, it is not currently met. More attention towards effective dietary strategies to reduce disease and mortality risk is imperative.

A plausible mechanism for our findings could be that non-Alzheimer's dementia and atherosclerotic cardiovascular disease share pathogenesis through atherosclerosis. In this study, individuals with low adherence to dietary guidelines were characterised by an atherogenic lipid profile with high levels of LDL cholesterol, non-HDL cholesterol and plasma triglycerides as a marker of triglyceride-rich lipoproteins, all causally related to atherosclerotic cardiovascular disease (Nordestgaard and Varbo, 2014; Ference *et al*., 2017; Mach *et al*., 2020). This lipid profile is most probably caused by primarily using saturated fat, eating more fast food, eating more sweets and fewer fruits and vegetables. Consistent evidence from genetic studies, prospective cohort studies, Mendelian randomisation studies and randomised controlled trials demonstrate that LDL causes atherosclerotic cardiovascular disease (Ference *et al*., 2017). LDL is the most abundant atherogenic lipoprotein in plasma and a key deliverer of cholesterol to the arterial wall (Borén *et al*., 2020). The likelihood of LDLs being kept inside the arterial intima leading to the establishment of atherosclerotic plaques advances when concentrations of circulating LDL particles increase (Skålén *et al*., 2002). Retention and subsequent modification of the LDL particle can elicit inflammatory processes and oxidative changes which influence the rate of plaque growth and the tendency to plaque disruption (Borén *et al*., 2020). Furthermore, due to their cholesterol content, triglyceride-rich lipoproteins are causally associated with atherosclerotic cardiovascular disease, as shown repeatedly in observational and genetic studies (Nordestgaard and Varbo, 2014; Hegele *et al*., 2020). When triglyceride concentrations are mild to moderately elevated (2–10 mmol/L), triglyceride-rich lipoproteins are sufficiently small to go through the arterial wall and therefore prone to preferential entrapment, and consequently accumulation causing atherosclerosis (Nordestgaard and Varbo, 2014). Our findings are supported by a recent report showing an association between moderate hypertriglyceridaemia and increased risk of non-Alzheimer's dementia in the general population (Nordestgaard *et al*., 2021). Importantly, in the present study we also show that the increasingly higher risk of non-Alzheimer's dementia with non-adherence to dietary guidelines was not observed in individuals on lipid-lowering therapy, indirectly suggesting that statins potentially may be used to prevent non-Alzheimer's dementia. Due to the observational nature of our study, these findings need to be tested in a randomised controlled trial before any conclusion on treatment benefits can be drawn. Other possible mechanisms for the present findings include the hypothesised effects of oxidative stress and neuroinflammation on risk of dementia (Kinney *et al*., 2018; Luo *et al*., 2020). Several risk factors including cardiovascular factors and metabolic diseases such as diabetes, often caused by unhealthy diets, are correlated with an immune response, also in the brain (Kinney *et al*., 2018). Systemic inflammation can lead to damage of the blood–brain barrier (BBB) which allows entry of immune cells into the brain. Moreover, impaired integrity of the BBB and thus increased BBB permeability can lead to excessive activation of microglial cells, which as a result release proinflammatory and reactive oxygen species.

Limitations of our study include the assessment of diet which was self-reported. Diets often change over time secondary to medications, influences from surroundings or diagnosis of a medical condition. However, when we analysed a subset of individuals who both participated at baseline and at follow-up examinations and fulfilled the FFQ at both visits, we found that the majority of individuals remained in the same group of adherence to dietary guidelines. Only a small fraction (<1%) changed dietary adherence group from either high to low or low to high. This indicates that our dietary instrument is a valid exposure over time. The FFQ did not take all national dietary advice into account, as for instance intake of dietary fibre or portion sizes, which therefore prevents us from investigating the impacts of macronutrients or adjusting for energy intake. However, the included questions have previously shown to be a sufficient proxy for an individual's dietary habits (Ewers *et al*., 2021). Furthermore, a previous Danish study compared an extensive FFQ of 198 items with a short FFQ, like the one in this study (Toft *et al*., 2007). They found that the short FFQ reflected dietary quality adequately and could be used as a tool to classify dietary patterns. Whether a change in adherence to dietary guidelines either to the better or worse was associated with Alzheimer's disease or non-Alzheimer's dementia could not be determined due to a limited number of cases in the subset. This important issue warrants further investigation in future prospective studies with repeated assessment of diet during follow-up. Non-response bias cannot be excluded, as individuals who did not respond to the FFQ, and thus excluded from this study, were less healthy and had lower socioeconomic status than those who did respond. This could limit the generalisability of the study. Study participants were white and from an ethnically homogenous population, which may also limit the generalisability of our findings. However, since the Danish national dietary guidelines are similar to guidelines of other countries, we would expect that our findings apply to other populations (Wilson *et al*., 2019; Mach *et al*., 2020). Finally, our findings for those treated with lipid-lowering therapy need to be confirmed in independent studies, preferably in randomised controlled trials. Strengths of the present study are addressed in the online Supplementary materials.

Low adherence to national dietary guidelines is associated with an atherogenic lipid profile and with an increased risk of non-Alzheimer's dementia – the subtype of dementia with a high frequency of vascular risk factors. The present study suggests that implementation of national dietary guidelines associated with an anti-atherogenic lipid profile could be of importance for prevention of non-Alzheimer's dementia and for improved cognitive health in the general population.

## Data Availability

Danish Law does not allow transfer of these data. Upon reasonable request to the corresponding author, the steering committee of the CGPS will evaluate whether data access through direct collaboration can be granted.

## Supplementary material

For supplementary material accompanying this paper visit https://doi.org/10.1017/S2045796022000567. click here to view supplementary material
