# Supplementary material for: Adherence to dietary guidelines and risk of dementia: a prospective cohort study of 94 184 individuals
Source: Epidemiol Psychiatr Sci. 2022 Oct 10;31:e71. doi: 10.1017/S2045796022000567 (PMC9583631; doi:10.1017/S2045796022000567)
Supplement: Supplementary file 1 [file S2045796022000567sup001.docx]

**Supplementary material**

**Adherence to national dietary guidelines and risk of dementia:**

**a prospective cohort study of 94,184 individuals**

Emilie W. Kjeldsen, Jesper Q. Thomassen, Katrine L. Rasmussen, Børge G. Nordestgaard, Anne Tybjærg-Hansen, Ruth Frikke-Schmidt

Table of contents

[Methods 3](#_Toc110945215)

[Clinical endpoints 3](#_Toc110945216)

[Laboratory analyses and genotyping 4](#_Toc110945217)

[Covariates 4](#_Toc110945218)

[Statistical analyses 5](#_Toc110945219)

[Results 8](#_Toc110945220)

[Discussion 8](#_Toc110945221)

[References 9](#_Toc110945222)

[Supplementary Tables 11](#_Toc110945223)

[Supplementary Table 1. Baseline characteristics for individuals included and excluded from the study. 11](#_Toc110945224)

[Supplementary Table 2. Food Frequency Questionnaire as a part of the extensive questionnaire in the Copenhagen General Population Study. 12](#_Toc110945225)

[Supplementary Table 3. Details of dietary food components 13](#_Toc110945226)

[Supplementary Table 4. Number of individuals with missing and imputed covariates in the Copenhagen General Population Study. 14](#_Toc110945227)

[Supplementary Table 5. Dietary patterns in CGPS and CGPS2 duplicates. 15](#_Toc110945228)

[Supplementary Figures 16](#_Toc110945229)

[Supplementary Figure 1. Study design. 16](#_Toc110945230)

[Supplementary Figure 2. Participant Flow Chart for the Copenhagen General Population Study. 17](#_Toc110945231)

[Supplementary Figure 3. Risk of non-Alzheimer’s dementia and Alzheimer’s disease according to dietary groups in 93,021 individuals in the Copenhagen General Population Study, excluding individuals with less than two years of follow-up. 18](#_Toc110945232)

[Supplementary Figure 4. Risk of non-Alzheimer’s dementia and Alzheimer’s disease according to dietary groups in 94,184 individuals in the Copenhagen General Population Study, adjusting additionally for ischemic heart disease and ischemic cerebrovascular disease. 19](#_Toc110945233)

[Supplementary Figure 5. Risk of non-Alzheimer’s dementia and Alzheimer’s disease according to dietary groups in 94,184 individuals in the Copenhagen General Population Study; imputed variables based on all covariates and endpoints. 20](#_Toc110945234)

[Supplementary Figure 6. Risk of cardiovascular mortality and all-cause mortality according to dietary groups in 94,184 individuals from the Copenhagen General Population Study - a positive control. 21](#_Toc110945235)

[Supplementary Figure 7. Interaction between level of adherence to dietary guidelines and covariates on risk of non-Alzheimer’s dementia in 94,184 individuals in the Copenhagen General Population Study. 22](#_Toc110945236)

# Methods

## Clinical endpoints

The national Danish Civil Registration System provided information on births, deaths, emigrations, and immigrations. Diagnosis codes were drawn from the national Danish Patient Registry and the national Danish Causes of Death Registry. The national Danish Patient Registry contains information on every patient contact from all clinical hospital departments since 1977, including emergency wards and outpatient clinics since 1994. Doctors in hospitals and general practitioners register the causes of every death in Denmark, which are then provided to the national Danish Causes of Death Registry. Information regarding death from cardiovascular diseases (International Classification of Disease (ICD)-8 codes 390–458 and ICD-10 codes I00–I99) was obtained from the Danish Register of Causes of Death censored on 31 December 2016. Information on cardiovascular death was therefore not available for people dying after 31 December 2016, whereas data on all-cause mortality were available up until 13 December 2018. If one of the top three ranked causes of death was cardiovascular, then the cause of death was considered of cardiovascular origin.

Alzheimer’s disease was ICD-8 code 290.10 and ICD-10 codes F00 and G30. Non-Alzheimer’s dementia was defined as vascular dementia (ICD10 code F01) and unspecified dementia (ICD8 code 290.18 and ICD10 code F03). Specifically, non-Alzheimer’s dementia did not include Lewy Body Dementia (ICD10 code G31.83) or frontotemporal dementia (ICD10 G31.0). The quality of these registry-based dementia diagnoses has previously been validated, (Phung *et al.*, 2007) and have been further validated in the Copenhagen General Population Study by the presence of the well-established apolipoprotein E (*APOE*) ε4 Alzheimer’s disease risk allele (Rasmussen *et al.*, 2015, 2019, 2020; Nordestgaard *et al.*, 2017; Kjeldsen *et al.*, 2018; Juul Rasmussen *et al.*, 2020).

## Laboratory analyses and genotyping

All blood samples were taken and analysed at baseline when individuals were included in the study. Total cholesterol, high-density lipoprotein (HDL) cholesterol, and triglycerides were measured in the non-fasting state using colorimetric assays (Konelab and Roche), and low-density lipoprotein (LDL) cholesterol was calculated using the Friedewald equation when triglycerides were below 4 mmol/L (354 mg/dL) or otherwise measured directly (Konelab and Roche) (Friedewald, Levy and Fredrickson, 1972; Nordestgaard *et al.*, 2016). In individuals using lipid-lowering therapy, values for total cholesterol, LDL cholesterol, and triglycerides were multiplied by 1.16 (=1/(1-0.14)), 1.23 (=1/1-0.19) and 1.12 (=1/(1-0.11)), respectively, corresponding to average reductions of 14%, 19% and 11% using common statin treatment regimens (Collins *et al.*, 2002; Kjeldsen *et al.*, 2022). Non-HDL cholesterol was total cholesterol minus HDL cholesterol. *APOE* genotype was determined as previously described and was categorized as ε22, ε32, ε42, ε33, ε43, and ε44 (Rasmussen *et al.*, 2015, 2019, 2020; Nordestgaard *et al.*, 2017; Kjeldsen *et al.*, 2018; Juul Rasmussen *et al.*, 2020).

## Covariates

Covariates were assessed at baseline. Household income, education, smoking, alcohol consumption, physical activity, and use of lipid-lowering therapy were all self-reported and categorical. Low household income was defined as <400,000 DKK/year (<53,800€/year). Smoking was cumulative in pack-years either as a continuous covariate or as a categorical covariate (0 pack-years, >0-≤10 pack-years, >10-≤20 pack-years or >20 pack-years). High alcohol consumption was >14/21 units of alcohol per week for women/men (1unit alcohol~12 g). Physical inactivity was ≤4 hours weekly of light physical exercise in leisure time. Lipid-lowering therapy was mainly statins (yes/no). Diabetes mellitus was self-reported disease, registered disease and/or hospitalization due to diabetes before study entry (ICD-8 code 249, 250; ICD-10 code E10-11, E13-14), medication prescribed for diabetes, and/or non-fasting plasma glucose levels of more than 11 mmol/L (>198mg/dl). Hypertension was systolic blood pressure ≥140 mm Hg, diastolic blood pressure ≥90 mm Hg, and/or self-reported use of antihypertensive medication. Systolic blood pressure was measured at examination and body mass index was calculated from measured weight in kilograms divided by measured height in meters squared, and both were treated as continuous covariates. These covariates were chosen as they are either associated with the exposure or the outcome. The cut-offs were primarily chosen based on recommendations from Danish health authorities or chosen to maintain evenly sized groups. Dichotomisation was preferred to sustain maximum power.

## Statistical analyses

We used Stata/SE version 17.0 (Stata Corp, College Station, TX). Probability values <0.001 are given as powers of 10. We used the command “stpower logrank” in Stata to calculate power; we had 80% statistical power at a two-sided P<0.05 to detect a hazard ratio of 1.37 for non-Alzheimer’s dementia and 1.22 for Alzheimer’s disease per increase in group of adherence to dietary guidelines. These calculations were based on the number of dementia cases, median follow-up time, and frequency of individuals in each exposure group. Kruskal-Wallis test or Cuzick’s test for trend were used to compare continuous covariates. Missing data on covariates (<1.28%) were imputed from age and sex using multinomial logistic regression for categorical variables and linear regression for continuous variables (Supplementary Table 4). Ten datasets were created, and results were synthesized from one extracted dataset. Imputation on all covariates and endpoints was used in a sensitivity analysis. The multiple imputed dataset (from age and sex) was used for the primary analyses, however, if only individuals with complete data were included, results were similar. No genotypes or endpoints were imputed.

The associations of dietary groups with non-Alzheimer’s dementia, Alzheimer’s disease, cardiovascular mortality, and all-cause mortality were examined using cause-specific Cox proportional hazards regression with age as underlying timescale (age adjustment) with delayed entry (left truncation at study examination), and with censoring at event, emigration, death, or end of follow-up. Model adjustments were done in three steps: a simple model including adjustment for age and sex; a second model adjusting for age, sex, household income, education, pack-years, alcohol consumption, physical activity, body mass index, diabetes, hypertension, lipid-lowering therapy, LDL cholesterol, HDL cholesterol, and triglycerides; and a third model adjusting for the second model plus ε2/ε3/ε4 *APOE* genotype. To test whether reverse causation affected our results, we excluded individuals with less than two years of follow-up in sensitivity analysis. Furthermore, we also conducted sensitivity analyses adjusting for ischemic heart disease and ischemic cerebrovascular disease as well as an analysis using imputed variables based on all covariates and endpoints.

There was no suspicion of nonproportionality, influence of outliers, or non-linearity of continuous covariates when checking cox proportional hazards assumptions. The analyses included checking for proportionality of hazards, linearity of effects, and absence of influential observations by plotting –ln(-ln[survival]) versus ln(analysis time), by Martingale residuals, and by deviance residuals, respectively.

To examine interactions between diet groups and each covariate on risk of non-Alzheimer’s dementia, we categorized each covariate into two groups. We tested for interactions by using an interaction term (diet group x covariate in two groups) in a model adjusted for age, sex, income, and education. P-values for interactions were by likelihood-ratio test comparing the model with and without the interaction term. Hazard ratios (HRs) per one unit increase in non-adherence to dietary guidelines were calculated using Cox proportional hazards regression models and shown for each group of the potentially interacting covariate.

# Results

Two thousand eighty-six individuals developed any type of dementia including 701 non-Alzheimer’s dementia cases and 1,385 Alzheimer’s disease cases. Follow-up time began at study inclusion and ended at occurrence of event, death (n=9,159), emigration (n=388) or on 13 December 2018. Median follow-up time was 9.0 years (range = <1–15 years) for any dementia. No individuals were lost to follow-up because of the complete Danish registries.

# Discussion

Strengths of the present study encompass the large cohort size and prospective design where dietary assessment and biochemical analyses precede a clinical diagnosis of dementia. Further, the Danish registries ensures no losses to follow-up and full information on deaths and emigrations. Additionally, the food frequency questionnaire has previously been validated, (Ewers *et al.*, 2021) and we now repeat and confirm this on updated endpoints and in larger dietary categories underscoring the robustness of the dietary exposure instrument. This enables us to use it as a powerful instrument similar to standardized diets such as the Mediterranean diet, the DASH, or the MIND (Pistollato *et al.*, 2018). Our approach focused on very similar dietary aspects as these regimes. The dietary assessment in our study was simple and focused only on the most important issues of food-based dietary guidelines ensuring feasibility. Finally, the core clinical criteria for Alzheimer’s disease have provided solid diagnostic accuracy in most patients, (McKhann *et al.*, 2011) and the quality of dementia diagnoses from the Danish registries have previously been validated (Phung *et al.*, 2007), with further validation in our study cohort by the presence of the recognised association with the *APOE* ɛ4 allele (Rasmussen *et al.*, 2015).

# References

**Collins R**, **Armitage J**, **Parish S**, **Sleight P** and **Peto R** (2002) MRC/BHF Heart Protection Study of cholesterol lowering with simvastatin in 20 536 high-risk individuals: A randomised placebo-controlled trial, *The Lancet* **360**, 7–22.

**Ewers B**, **Marott JL**, **Schnohr P**, **Nordestgaard BG** and **Marckmann P** (2021) Non-adherence to established dietary guidelines associated with increased mortality: The Copenhagen General Population Study, *European Journal of Preventive Cardiology* **28**, 1259–1268.

**Friedewald WT**, **Levy RI** and **Fredrickson DS** (1972) Estimation of the concentration of low-density lipoprotein cholesterol in plasma, without use of the preparative ultracentrifuge., *Clinical Chemistry* **18**, 499–502.

**Juul Rasmussen I**, **Rasmussen KL**, **Nordestgaard BG**, **Tybjærg-Hansen A** and **Frikke-Schmidt R** (2020) Impact of cardiovascular risk factors and genetics on 10-year absolute risk of dementia: risk charts for targeted prevention, *European Heart Journal* **41**, 4024–4033.

**Kjeldsen EW**, **Thomassen JQ**, **Rasmussen KL**, **Nordestgaard BG**, **Tybjærg-Hansen A** and **Frikke-Schmidt R** (2022) Impact of diet on ten-year absolute cardiovascular risk in a prospective cohort of 94 321 individuals: A tool for implementation of healthy diets, *The Lancet Regional Health - Europe* **19**, 100419.

**Kjeldsen EW**, **Tybjaerg-Hansen A**, **Nordestgaard BG** and **Frikke-Schmidt R** (2018) ABCA7 and risk of dementia and vascular disease in the Danish population, *Annals of Clinical and Translational Neurology* **5**, 41–51.

**McKhann G**, **Knopman DS**, **Chertkow H**, **Hymann B**, **Jack CR**, **Kawas C**, **Klunk W**, **Koroshetz W**, **Manly J**, **Mayeux R**, **Mohs R**, **Morris J**, **Rossor M**, **Scheltens P**, **Carrillo M**, **Weintrub S** and **Phelphs C** (2011) The diagnosis of dementia due to Alzheimer’s disease: Recommendations from the National Institute on Aging-Alzheimer’s Association workgroups on diagnostic guidelines for Alzheimer’s disease, *Alzheimer’s & Dementia* **7**, 263–269.

**Nordestgaard BG**, **Langsted A**, **Mora S**, **Kolovou G**, **Baum H**, **Bruckert E**, **Watts GF**, **Sypniewska G**, **Wiklund O**, **Borén J**, **Chapman MJ**, **Cobbaert C**, **Descamps OS**, **Von Eckardstein A**, **Kamstrup PR**, **Pulkki K**, **Kronenberg F**, **Remaley AT**, **Rifai N**, **Ros E** and **Langlois M** (2016) Fasting is not routinely required for determination of a lipid profile: Clinical and laboratory implications including flagging at desirable concentration cut-points - A joint consensus statement from the European Atherosclerosis Society and European Fede, *European Heart Journal* **37**, 1944–1958.

**Nordestgaard LT**, **Tybjærg-Hansen A**, **Nordestgaard BG** and **Frikke-Schmidt R** (2017) Body mass index and risk of Alzheimer’s disease: A Mendelian randomization study of 399,536 individuals, *The Journal of Clinical Endocrinology & Metabolism* **102**, 2310–2320.

**Phung TKT**, **Andersen BB**, **Høgh P**, **Kessing LV**, **Mortensen PB** and **Waldemar G** (2007) Validity of dementia diagnoses in the Danish hospital registers, *Dementia and Geriatric Cognitive Disorders* **24**, 220–228.

**Pistollato F**, **Iglesias RC**, **Ruiz R**, **Aparicio S**, **Crespo J**, **Lopez LD**, **Manna PP**, **Giampieri F** and **Battino M** (2018) Nutritional patterns associated with the maintenance of neurocognitive functions and the risk of dementia and Alzheimer’s disease: A focus on human studies, *Pharmacological Research* **131**, 32–43.

**Rasmussen KL**, **Tybjærg-Hansen A**, **Nordestgaard BG** and **Frikke-Schmidt R** (2015) Plasma levels of apolipoprotein E and risk of dementia in the general population, *Annals of Neurology* **77**, 301–311.

**Rasmussen KL**, **Tybjærg-Hansen A**, **Nordestgaard BG** and **Frikke-Schmidt R** (2019) Plasma levels of apolipoprotein E, APOE genotype, and all-cause and cause-specific mortality in 105 949 individuals from a white general population cohort, *European Heart Journal* **40**, 2813–2824.

**Rasmussen KL**, **Tybjærg-Hansen A**, **Nordestgaard BG** and **Frikke-Schmidt R** (2020) APOE and dementia – resequencing and genotyping in 105,597 individuals, *Alzheimer’s and Dementia* **16**, 1624–1637.

# Supplementary Tables

## Supplementary Table 1. Baseline characteristics for individuals included and excluded from the study.

Values are mean (±standard deviation) or percentage and are from the day of enrolment. Please see methods (covariates) for details.

| **Baseline characteristics** | **Included individuals** | **Excluded individuals** | **P-value** |
| --- | --- | --- | --- |
| Number | 94,184 | 13,904 |  |
| Age, years | 58.0 (±13.0) | 58.2 (±13.7) | 0.39 |
| Men, no. (%) | 42,464 (45) | 6,053 (44) | 0.003 |
| Low household income, no. (%) | 33,364 (35) | 5,819 (42) | <0.001 |
| Education <8 years, no. (%) | 8,782 (9) | 1,743 (13) | <0.001 |
| Body mass index, kg/m^2^ | 26.1 (±4.3) | 26.3 (±4.3) | <0.001 |
| Physical inactivity in leisure time, no. (%) | 45,175 (48) | 7,089 (51) | <0.001 |
| Cumulative tobacco consumption, pack-years | 11.8 (±18.2) | 13.7 (±19.7) | <0.001 |
| High alcohol consumption, no. (%) | 16,215 (17) | 2,431 (17) | 0.60 |
| Hypertension, no. (%) | 56,743 (60) | 8,391 (60) | 0.86 |
| Diabetes mellitus, no. (%) | 3,937 (4) | 646 (5) | 0.37 |
| Lipid-lowering therapy, no. (%) | 11,485 (12) | 1,558 (11) | 0.06 |

#

## Supplementary Table 2. Food Frequency Questionnaire as a part of the extensive questionnaire in the Copenhagen General Population Study.

| **1** | **How many main meals do you eat per day?** | | | | | | | | | | | | | |
| --- | --- | --- | --- | --- | --- | --- | --- | --- | --- | --- | --- | --- | --- | --- |
|  | Amount: | | | | | | | | | | | | | |
| **2** | **How many slices of bread do you normally eat in a day?** | | | | | | | | | | | | | |
|  | Amount: | | | | | | | | | | | | | |
| **3** | **What type of fat do you most often use on bread? (Please, only mark with one cross)** | | | | | | | | | | | | | |
|  | Nothing Butter Butter blend Plant margarine Minarine Other | | | | | | | | | | | | | |
|  |  | | | | | | | | | | | | | |
| **4** | **How many times a week do you eat the type of cold cuts mentioned below?** | | | | | | | | | | | | | |
|  | Cold meat cuts Pate Cold fish cuts Cheese | | | | | | | | | | | | | |
|  |  | | | | | | | | | | | | | |
| **5** | **How many times a week do you eat the type of warm foods mentioned below?** | | | | | | | | | | | | | |
|  | Beef/veal Pork Poultry Fish Fast food | | | | | | | | | | | | | |
|  |  | | | | | | | | | | | | | |
| **6** | **What type of fat do you most often use when preparing a warm meal? (Please, only mark with one cross)** | | | | | | | | | | | | | |
|  | Nothing | Butter | | Butter blend | | Frying margarine | | | Plant margarine | | Minarine | Cooking oil | | Other |
|  |  | | | | | | | | | | | | | |
| **7** | **How often do you eat vegetables as a part of a snack, breakfast, lunch or as a bigger part of a warm meal? (Please, only mark with one cross)** | | | | | | | | | | | | | |
|  | Almost never | | 1-3/month | | 1-2/month | | 2-3/week | | | 5-6/week | 1/day | 2-3/day | >3/day | |
|  |  | | | | | | | | | | | | | |
| **8** | **How often do you eat fruit as a whole piece of fruit or as a portion of fruit? (Please, only mark with one cross)** | | | | | | | | | | | | | |
|  | Almost never | | 1-3/month | | 1-2/month | | 2-3/week | | | 5-6/week | 1/day | 2-3/day | >3/day | |
|  |  | | | | | | | | | | | | | |
| **9** | **What amount of the following beverages do you drink per week?** | | | | | | | | | | | | | |
|  | Coca Cola x ½ L | | | | | | | Other soft drinks x ½ L | | | | | | |
|  |  | | | | | | | | | | | | | |

The food frequency questionnaire (FFQ) was filled out by each individual at baseline. The extensive self-administered questionnaire, including the FFQ, was reviewed together with an investigator on the day of attendance (baseline; inclusion in the study). Questions that were not included in the dietary assessment are grey.

## Supplementary Table 3. Details of dietary food components

| **Question priority** | **Dietary food component** | **In agreement**  **with guidelines** | **In disagreement**  **with guidelines** |
| --- | --- | --- | --- |
| A | Fats for cold meals | Unsaturated^a^ | Saturated^c^ |
| A | Fats for warm meals | Unsaturated^b^ | Saturated^d^ |
| A | Vegetables, weekly servings | >3 | <2 |
| A | Fruit, weekly servings | >3 | <2 |
| B | Fish, weekly servings | >3 | <1 |
| B | Sugar-sweetened beverages, L/week | <0.5 | >1 |
| C | Cold meat cuts,^e^ weekly servings | <5 | >7 |
| C | Fast food,^f^ weekly servings | <1 | >1 |

^a^Unsaturated fat spreads defined as soft margarines or no use of fat spread.

^b^Unsaturated fat for cooking defined as vegetable oils or no use of fat for cooking.

^c^Saturated fat spread, i.e. butter, butter-based blends.

^d^Saturated fat for cooking, i.e. butter, butter-based blends or hard margarines.

^e^Cold meat cuts such as sausages and pâtés for open sandwiches.

^f^Fast food including food purchased at hot dog stands, grills, shawarma restaurants, burger chains and take away pizza.

## Supplementary Table 4. Number of individuals with missing and imputed covariates in the Copenhagen General Population Study.

| Covariate | Complete | Imputed (N) | Imputed (%) | Total |
| --- | --- | --- | --- | --- |
| Age | 94,184 | 0 | 0.00% | 94,184 |
| Sex | 94,184 | 0 | 0.00% | 94,184 |
| Household income | 93,151 | 1,033 | 1.10% | 94,184 |
| Educational level | 93,967 | 217 | 0.23% | 94,184 |
| Body mass index | 94,045 | 139 | 0.15% | 94,184 |
| Physical activity in leisure time | 93,641 | 543 | 0.58% | 94,184 |
| Smoking status | 93,947 | 237 | 0.25% | 94,184 |
| Alcohol consumption | 94,177 | 7 | 0.01% | 94,184 |
| Hypertension | 94,162 | 22 | 0.02% | 94,184 |
| Diabetes mellitus | 94,181 | 3 | 0.00% | 94,184 |
| Lipid lowering therapy | 93,900 | 284 | 0.30% | 94,184 |
| LDL cholesterol | 93,107 | 1,077 | 1.14% | 94,184 |
| HDL cholesterol | 93,351 | 833 | 0.88% | 94,184 |
| Triglycerides | 93,348 | 836 | 0.86% | 94,184 |

## Supplementary Table 5. Dietary patterns in CGPS and CGPS2 duplicates.

| **Adherence to dietary guidelines** | **CGPS** | | **CGPS2** | |
| --- | --- | --- | --- | --- |
|  | Frequency (N) | Percent (%) | Frequency (N) | Percent (%) |
| High | 2,484 | **19.41** | 2,663 | **20.80** |
| Intermediate | 9,178 | **71.70** | 8,840 | **69.06** |
| Low | 1,138 | **8.89** | 1,297 | **10.13** |
| Total | 12,800 | 100.00 | 12,800 | 100.00 |
|  |  |  |  |  |
| **Change in group of adherence** | Frequency (N) | Percent (%) |  |  |
| High 🡪 high | 1,039 | 8.12 |  |  |
| High 🡪 intermediate | 1,395 | 10.90 |  |  |
| High 🡪 low | 50 | 0.39 |  |  |
| Intermediate 🡪 high | 1,602 | 12.52 |  |  |
| Intermediate 🡪 intermediate | 6,841 | 53.45 |  |  |
| Intermediate 🡪 low | 735 | 5.74 |  |  |
| Low 🡪 high | 22 | 0.17 |  |  |
| Low 🡪 intermediate | 604 | 4.72 |  |  |
| Low 🡪 low | 512 | 4.00 |  |  |
| Overall no change | 8,382 | **65.48** |  |  |

The distribution of individuals in the three dietary categories was assessed in individuals both attending CGPS (recruitment between 2003-2015) and CGPS2 (follow-up started in 2015) to estimate changes in overall diets over time. Changes in dietary patterns between groups of adherence to dietary guidelines are shown in the bottom table. The mean time between baseline and second examination follow-up was 10 years.

# Supplementary Figures

## Supplementary Figure 1. Study design.

**
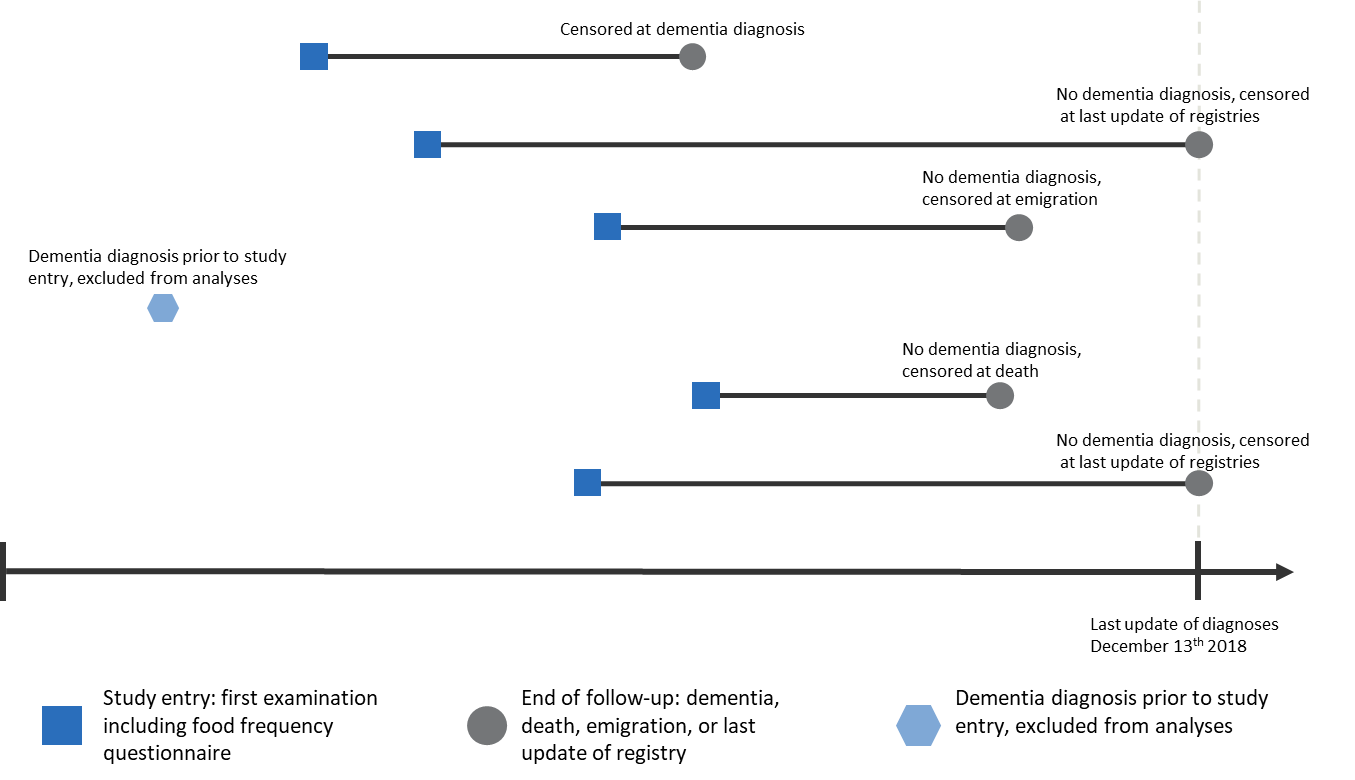
**

The figure illustrates six examples of follow-up time with censoring or exclusion of participants in the studies. Study entry of CGPS was at the time of the first examination (2003-2015) and included assessment of baseline covariates (blue square). Follow-up ended at occurrence of event (n=2,086), death (n=9,159), emigration (n=388), or on December 13^th^, 2018 (last update of the registry), whichever came first (grey circle). Individuals with event before study entry were not included in the study (light blue hexagon). CGPS=Copenhagen General Population Study.

## Supplementary Figure 2. Participant Flow Chart for the Copenhagen General Population Study.


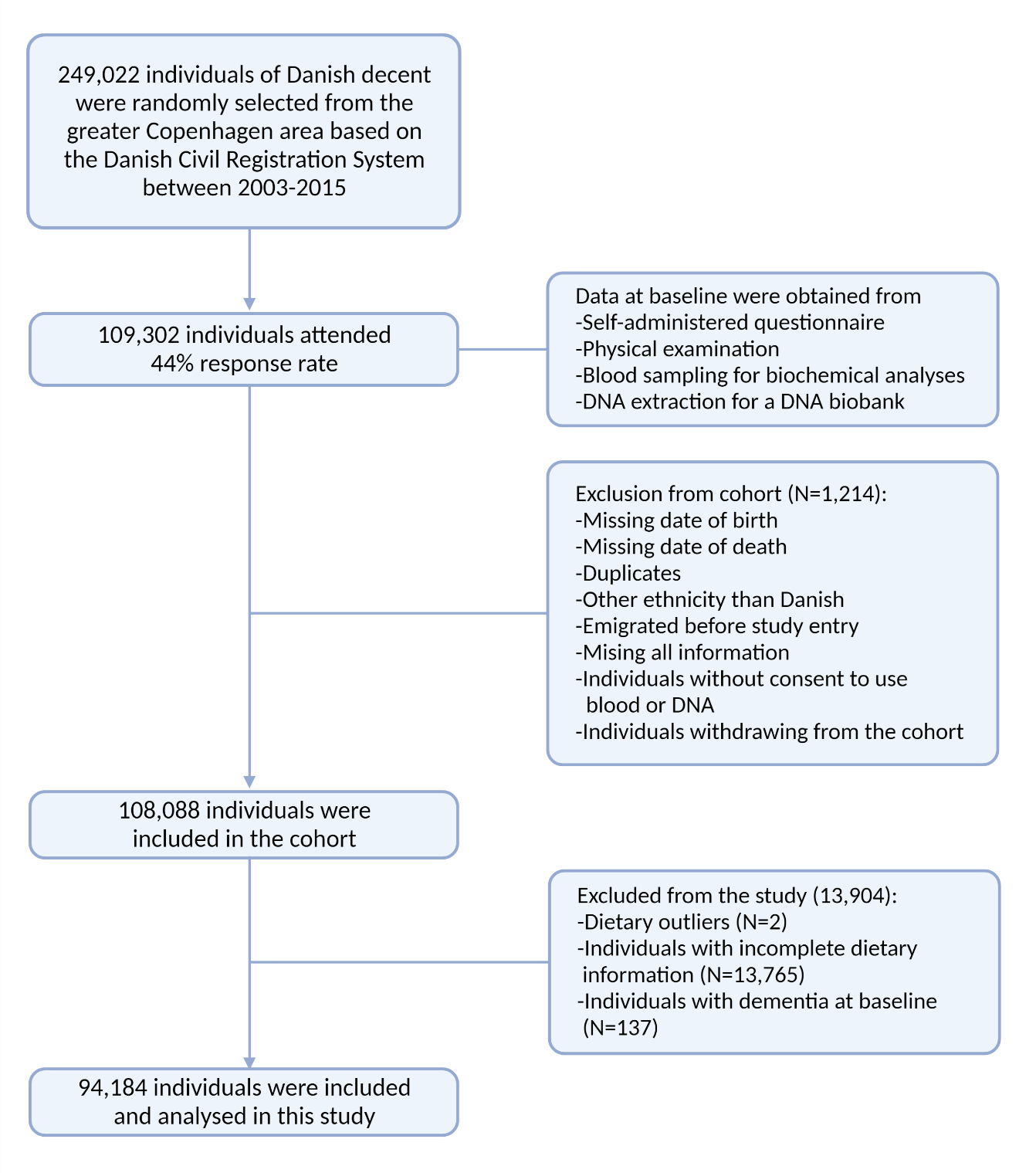


## Supplementary Figure 3. Risk of non-Alzheimer’s dementia and Alzheimer’s disease according to dietary groups in 93,021 individuals in the Copenhagen General Population Study, excluding individuals with less than two years of follow-up.


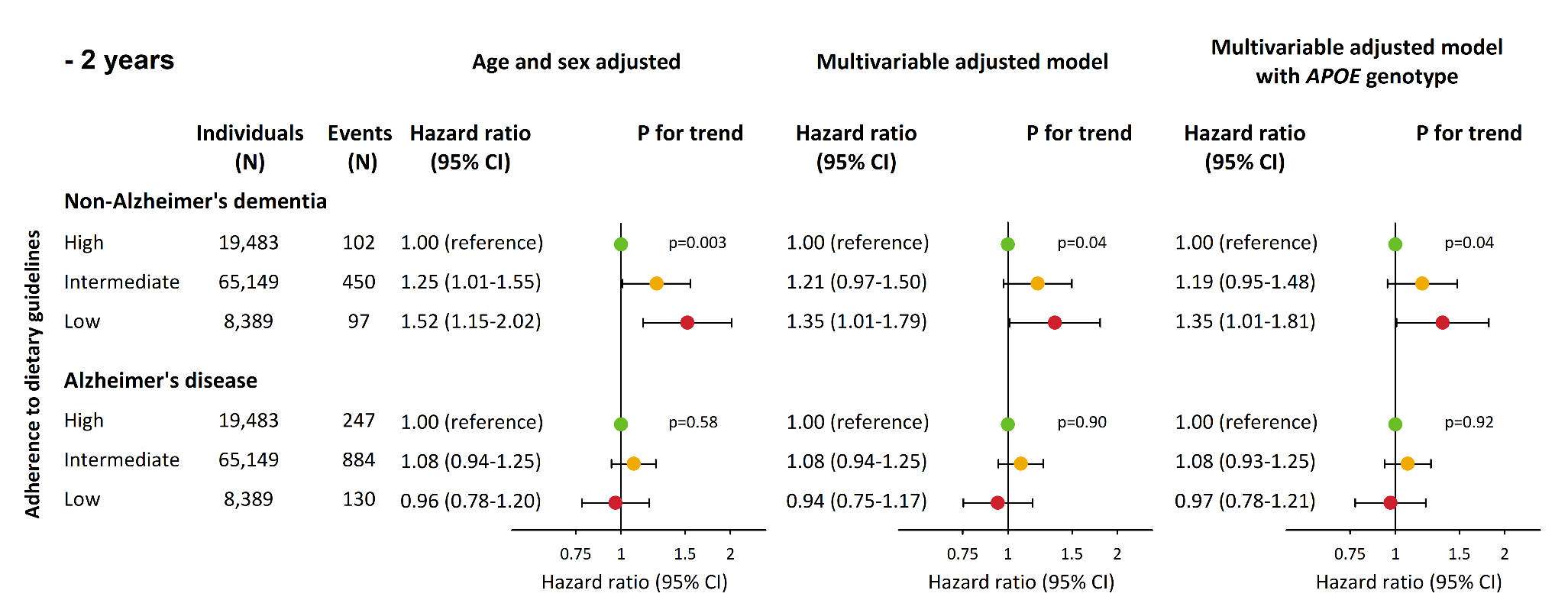


Multivariable adjustment was for age (as timescale), sex, household income, education, smoking, alcohol consumption, physical activity, body mass index, diabetes, hypertension, lipid-lowering therapy, LDL cholesterol, HDL cholesterol, and triglycerides. *APOE* genotype=apolipoprotein E ε2/ε3/ε4 genotype; CI=confidence interval; HDL cholesterol=high-density lipoprotein cholesterol; LDL cholesterol=low-density lipoprotein cholesterol.

## Supplementary Figure 4. Risk of non-Alzheimer’s dementia and Alzheimer’s disease according to dietary groups in 94,184 individuals in the Copenhagen General Population Study, adjusting additionally for ischemic heart disease and ischemic cerebrovascular disease.


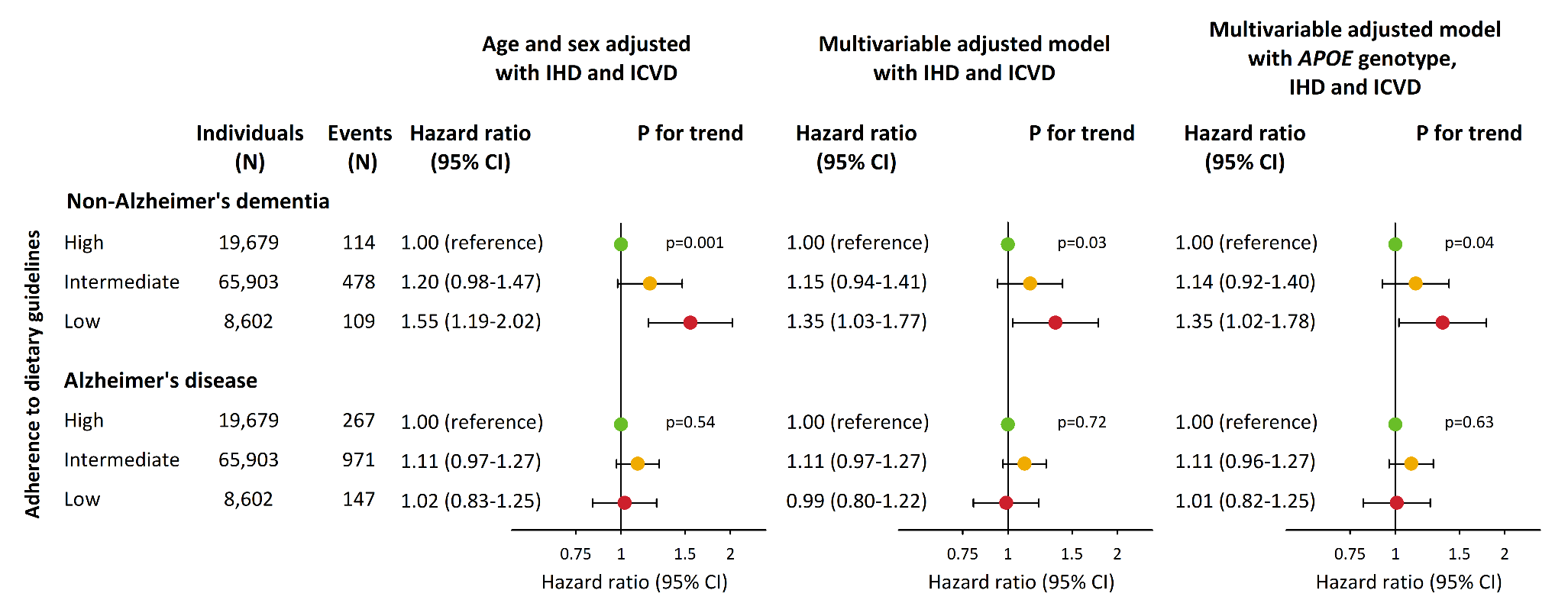


Multivariable adjustment was for age (as timescale), sex, household income, education, smoking, alcohol consumption, physical activity, body mass index, diabetes, hypertension, lipid-lowering therapy, LDL cholesterol, HDL cholesterol, triglycerides, IHD and ICVD prior to study entry. *APOE* genotype=apolipoprotein E ε2/ε3/ε4 genotype; CI=confidence interval; HDL cholesterol=high-density lipoprotein cholesterol; IHD=ischemic heart disease; ICVD=ischemic cerebrovascular disease; LDL cholesterol=low-density lipoprotein cholesterol.

## Supplementary Figure 5. Risk of non-Alzheimer’s dementia and Alzheimer’s disease according to dietary groups in 94,184 individuals in the Copenhagen General Population Study; imputed variables based on all covariates and endpoints.


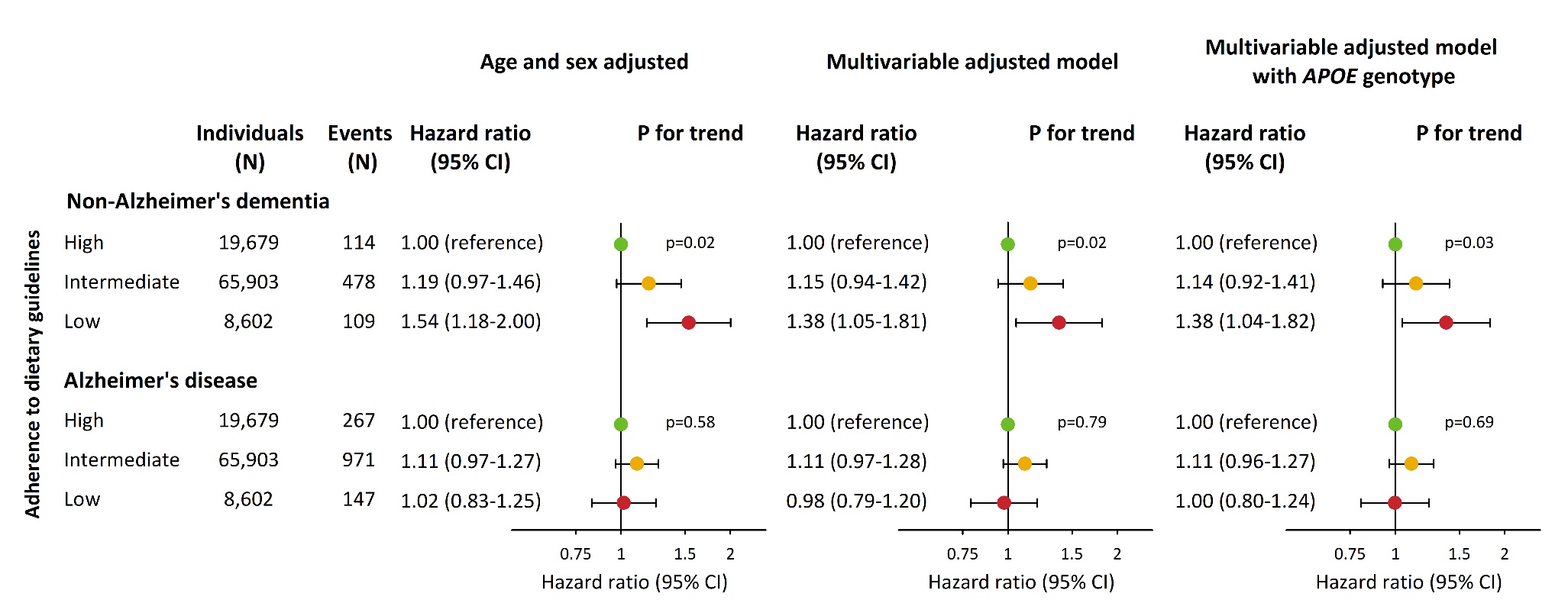


Multivariable adjustment was for age (as timescale), sex, household income, education, smoking, alcohol consumption, physical activity, body mass index, diabetes, hypertension, lipid-lowering therapy, LDL cholesterol, HDL cholesterol, triglycerides, IHD and ICVD prior to study entry. Variables were multiple imputed based on all covariates and endpoints included in the study. *APOE* genotype=apolipoprotein E ε2/ε3/ε4 genotype; CI=confidence interval; HDL cholesterol=high-density lipoprotein cholesterol; LDL cholesterol=low-density lipoprotein cholesterol.

## Supplementary Figure 6. Risk of cardiovascular mortality and all-cause mortality according to dietary groups in 94,184 individuals from the Copenhagen General Population Study - a positive control.


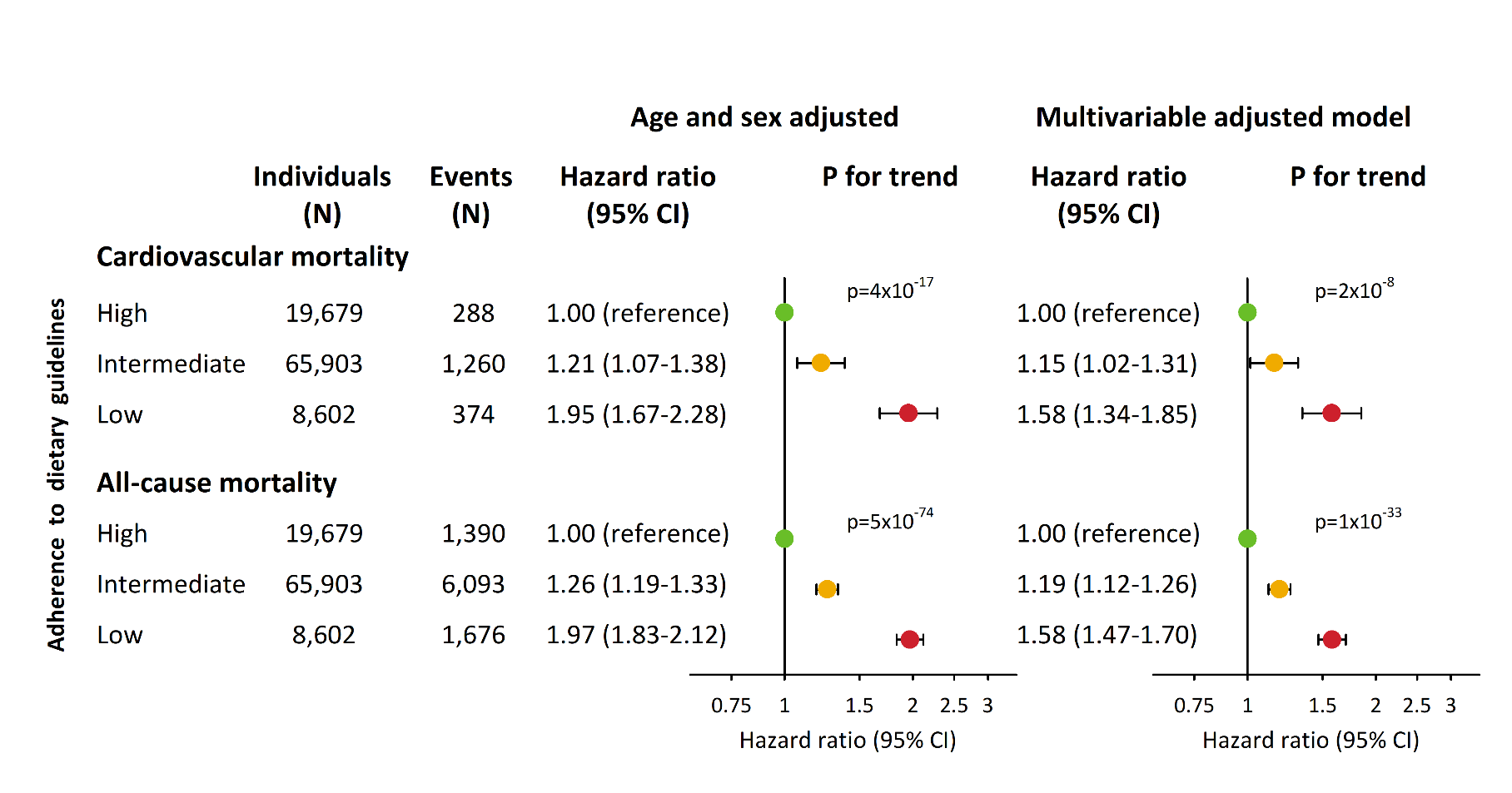


Multivariable adjustment was for age (as timescale), sex, household income, education, smoking, alcohol consumption, physical activity, body mass index, diabetes, hypertension, lipid-lowering therapy, LDL cholesterol, HDL cholesterol, and triglycerides. CI=confidence interval; HDL cholesterol=high-density lipoprotein cholesterol; LDL cholesterol=low-density lipoprotein cholesterol.

## Supplementary Figure 7. Interaction between level of adherence to dietary guidelines and covariates on risk of non-Alzheimer’s dementia in 94,184 individuals in the Copenhagen General Population Study.





Hazard ratios per one increase in group based on adherence to dietary guidelines for risk of non-Alzheimer’s dementia were adjusted for age (as timescale), sex, income and education. Household income (<400,000 DKK/year vs. ≥ 400,00 DKK/year), education (<8 years vs. ≥8 years), body mass index (≤50^th^ vs. >50^th^ percentile), physical activity (<4 hours/week vs. ≥4 hours/week), smoking in pack-years (≤50^th^ vs. >50^th^ percentile), alcohol (women/men 0-14/0-21 drinks/week vs. >15/>22 drinks/week), hypertension (yes vs. no), diabetes mellitus (yes vs. no), lipid-lowering therapy (yes vs. no), triglycerides (≤50^th^ vs. >50^th^ percentile), LDL cholesterol (≤50^th^ vs. >50^th^ percentile), HDL cholesterol (≤50^th^ vs. >50^th^ percentile). Interaction was tested by using two-factor interaction terms and a likelihood ratio test in the Cox regression model. P for interaction was calculated using likelihood ratio test. CI=confidence interval; HDL cholesterol=high-density lipoprotein cholesterol; HR=hazard ratio; LDL cholesterol=low-density lipoprotein cholesterol.
